# Double-Emulsion Copolyester Microcapsules for Sustained Intraperitoneal Release of Carboplatin

**DOI:** 10.3390/jfb10040055

**Published:** 2019-12-06

**Authors:** Aneta Cymbaluk-Płoska, Peter Sobolewski, Anita Chudecka-Głaz, Ewa Wiśniewska, Joanna Łapczuk, Marcin Frankowski, Marek Droździk, Miroslawa El Fray

**Affiliations:** 1 Department and Clinic of Gynecological Surgery and Gynecological Oncology of Adults and Adolescents, Pomeranian Medical University, ul. Powstanców Wlkp. 72, 70-111 Szczecin, Poland; aneta.cymbaluk@gmail.com (A.C.-P.); achudeckaglaz@icloud.com (A.C.-G.); 2 Department of Polymer and Biomaterials Science, Faculty of Chemical Technology and Engineering, West Pomeranian University of Technology, Szczecin, Al. Piastów 45, 70-311 Szczecin, Poland; psobolewski@zut.edu.pl (P.S.); ewaw@zut.edu.pl (E.W.); 3 Department of Experimental and Clinical Pharmacology, Pomeranian Medical University, ul. Powstanców Wlkp. 72, 70-111 Szczecin, Poland; joannalapczuk@googlemail.com (J.Ł.); marek.drozdzik@pum.edu.pl (M.D.); 4 Faculty of Chemistry, Adam Mickiewicz University in Poznań, ul. Uniwersytetu Poznańskiego 8, 61-614 Poznań, Poland; marcin.frankowski@amu.edu.pl

**Keywords:** controlled drug release, microencapsulation, carboplatin, ovarian cancer, intraperitoneal delivery

## Abstract

Despite on-going medical advances, ovarian cancer survival rates have stagnated. In order to improve IP delivery of platinum-based antineoplastics, we aimed to develop a sustained drug delivery system for carboplatin (CPt). Toward this aim, we pursued a double emulsion process for obtaining CPt-loaded microcapsules composed of poly(ethylene terephthalate-ethylene dilinoleate) (PET-DLA) copolymer. We were able to obtain PET-DLA microspheres in the targeted size range of 10–25 µm (median: 18.5 µm), to reduce intraperitoneal clearance by phagocytosis and lymphoid transit. Empty microspheres showed the lack of toxicity in vitro. The double emulsion process yielded 2.5% w/w CPt loading and obtained microcapsules exhibited sustained (>20 day) zero-order release. The encapsulated CPt was confirmed to be bioavailable, as the microcapsules demonstrated efficacy against human ovarian adenocarcinoma (SK-OV-3) cells in vitro. Following intraperitoneal injection in mice, we did not observe adhesions, only mild, clinically-insignificant, local inflammatory response. Tissue platinum levels, monitored over 14 days using atomic absorption spectroscopy, revealed low burst and reduced systemic uptake (plasma, kidney), as compared to neat carboplatin injection. Overall, the results demonstrate the potential of the developed microencapsulation system for long-term intraperitoneal sustained release of carboplatin for the treatment of ovarian cancer.

## 1. Introduction

Ovarian cancer accounted for >180,000 deaths worldwide in 2018, representing 4.4% of cancer related deaths in females [1]. The incidence rate is on the rise, increasing by 23% between 2015 and 2018, and predicted to exceed 370,000 by 2035, accounting for >250,000 deaths [2]. Shockingly, the 5-year survival worldwide ranges from 20–49%, depending on the country, and has remained largely flat since 1995, despite on-going medical advances [2]. This is likely due to the very high rate of recurrence: approx. 80% of patients relapse within 18 months [3]. The first-line standard of care for ovarian cancer involves surgery, with the aim of removing all macroscopic tumor, and chemotherapy combining paclitaxel and carboplatin serving as (neo)adjuvant therapy [4]. Because primary ovarian cancer, as well as its dissemination and recurrence are localized in the peritoneal cavity, local intraperitoneal (IP) delivery has long been explored as a means of improving outcomes and a recent meta-analysis [5] has demonstrated that IP delivery of platinum-based antineoplastics (platins: cisplatin, carboplatin) increases overall survival (hazard ratio of 0.81), but at the cost of more serious gastrointestinal (GI) complications, along with catheter-related complications. Protocols typically involve IP infusions of 2 L of drug in saline via catheter (single-use or implanted port and catheter system), every 21–28 days for 6–8 cycles. The reason for the GI complications is likely due to the small molecular size and high water solubility of platins, enabling rapid uptake to abdominal organs, as well as the circulation [6,7]. As a result, developing drug delivery systems (DDS) for ovarian cancer therapy is an attractive strategy towards improving outcomes, while reducing complications.

Based on the current standard of care, we decided to focus on an IP carboplatin microsphere DDS. Raavé et al. have recently published a systematic review [8] of numerous animal studies aimed at evaluating various DDS as potential ovarian cancer therapies. They conclude that platins may not be good candidates for ovarian cancer drug delivery systems. However, their review only included one microscale platin DDS, which relied on tumor cell-derived microparticles as the delivery vehicle [9], while the remaining studies were all nanoscale. While nanoparticle strategies to anticancer drug delivery have generated the most excitement, a recent literature survey [10] found that actual delivery of nanoparticles is very poor, with the median result being <1% of the dose delivered to tumor. Thus, given the peritoneal localization of primary ovarian cancer, as well as its dissemination and recurrence, a DDS approach centered on IP delivery of microparticles may be more promising. If the microparticles are sufficiently large (>10 µm), then their size will minimize both phagocytosis by peritoneal macrophages [11], as well as lymphoid transport [12], facilitating controlled release of the drug within the peritoneal cavity.

Microencapsulation is not a new technique [13] and the microencapsulation of platins has been an area of active investigation over the past 30+ years, particularly involving polymeric systems composed of polylactic acid (PLA) or poly(lactic-*co*-glycolic acid) (PLGA) [14,15,16,17,18,19,20,21,22,23,24]. However, all of these systems rely on a single emulsion process: solid platin is dispersed in a polymer solution serving as the organic phase, followed by emulsification in an aqueous dispersed phase. This yields a monolithic system with drug trapped within a polymer matrix. While this is a suitable process for hydrophobic drugs (e.g., paclitaxel), it is not ideal for hydrophilic ones, like platins, because the drug will not dissolve in the organic phase and will instead remain as solid particles or crystals. In these studies, the encapsulation amount of platin ranged from 4% w/w [18] to as high as 40% w/w [14], but in the latter case the drug was clearly present as crystals. In vitro release profiles were characterized by marked burst phases (typically 15–30%) and relatively short release times (from a few days to 2 weeks). Longer release times, up to 60 days were reported by Spenlehauer et al. [14], but in this case ~75% of the drug was released in the last 5 days, likely due to total capsule degradation/dissolution. An alternative approach using aqueous solutions of carboplatin and gelatin as the polymer [25], emulsified in paraffin, may be more appropriate for encapsulating the water soluble platin, but this system yielded very high burst (~40%) and very rapid release, with ~90% release over the first 5 h. A similar system, using lyophilized gelatin microspheres rehydrated with aqueous cisplatin has also been reported [26], but while this system was characterized by somewhat lower burst (>20%), the stability in vitro remained low (>60% degradation within 24 h).

To our knowledge, we are the only group pursuing a double emulsion process for obtaining platin-loaded microcapsules. Instead of dispersing solid carboplatin (CPt) in the organic phase, we prepare an emulsion of aqueous carboplatin in the organic phase, which is then dispersed in an aqueous continuous phase. The advantage of this approach is that it yields a diffusion-controlled reservoir system, rather than a monolithic system [27]. The double emulsion process can be expected to yield CPt trapped inside a polymer membrane, resulting in zero-order (constant flux) kinetics. In our previous work [28], we demonstrated the use of a double emulsion process to encapsulate carboplatin in poly(ester-amide)-PEG copolymer microcapsules. While we obtained the desired zero-order kinetics, the drug release was still too rapid, lasting less than 2 weeks, likely due to the amphiphilic nature of the polymer and relatively fast degradation. Here, we set to develop a microencapsulation system that would provide a much longer release time, while maintaining zero-order kinetics. Towards this aim, we selected an aromatic/aliphatic polyester copolymer with poly(ethylene terephthalate) (PET) sequences as hard segments and poly(ethylene dilinoleate) soft segments [29]. This copolymer family, poly(ethylene terephthalate-*co*-ethylene dilinoleate) (PET-DLA), was developed to serve as elastomeric biomaterials and their in vivo biocompatibility has already been demonstrated [30]. Importantly, the incorporation of ethylene dilinoleate soft segments increases the amount of amorphous phase, increasing susceptibility to hydrolytic degradation [29,31], however indicating relatively low mass loss and a bulk erosion mechanism for the PET-DLA materials. We hypothesized that by using the previously developed double emulsion system with this poly(aromatic/aliphatic-ester) that is more hydrophobic and stable, as compared to the poly(ester-amide)-PEG copolymer we used previously, we could obtain carboplatin-containing microspheres with greatly extended zero-order release kinetics. Here we first confirmed the ability to obtain PET-DLA microspheres of sufficient size, as well as their lack of toxicity in vitro. Next, we demonstrate the encapsulation of carboplatin in the PET-DLA microspheres and confirm prolonged (>20 day) zero-order carboplatin release, as well as efficacy against human ovarian adenocarcinoma cells in vitro. Finally, we present in vivo pharmacodynamics and biocompatibility following IP delivery to mice.

## 2. Results and Discussion

**Figure 1:**
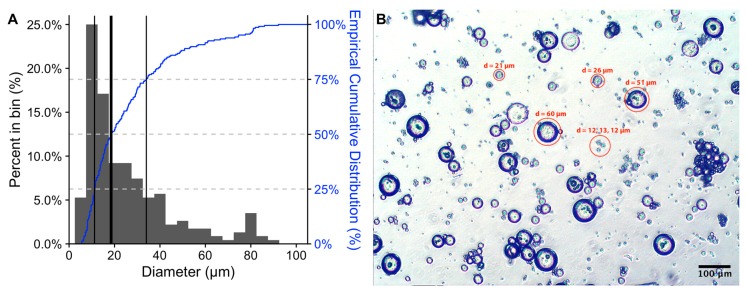
(**A**) Histogram (bin width = 5, n = 228) and empirical cumulative distribution (blue line, right y-axis) of diameters of microspheres loaded with CPt. The thick black bar indicates median: 18.5 µm, while the thin black bars represent quartiles: 1st 11.2 µm, 3rd 34.0 µm. (**B**) Representative micrograph of CPt-loaded microspheres (scale bar indicates 100 µm).

The size of the microspheres is very important factor that influences residence time in the peritoneum. In order to avoid phagocytosis by macrophages [11] and lymphoid transport [12], microspheres must be >10 µm in diameter. Therefore, it was our aim to prepare polymeric microspheres with diameters in the range of 10 to 20 μm. Using optical microscopy, we assessed microsphere size (Figure 1).

As can been seen, the majority of microspheres fall in the range of 10–25 µm, with the median diameter being 18.5 µm, the interquartile range being 22.8 µm, and span being 2.5. However, as can be seen from both the histogram and empirical cumulative distribution (Figure 1A), the primary diameter distribution has positive skew; a minor 2nd population (<10% of the total), centered on ~80 µm, was also observed. No differences were observed between CPt-loaded and empty microspheres and similar results were obtained for 4 different batches of microspheres. The microspheres smaller than 10 µm (~17.5% of the distribution) are undesirable, as they will likely be cleared from the peritoneum by either phagocytosis [11] or lymphoid transport [12]. However, while we targeted particles in the 10–20 µm range, it is not clear that particles larger than 20 µm are undesired, as they should remain in the peritoneum. In fact, Kohane et al. [32] report that larger microparticles, 20–60 µm in diameter, resulted in reduced adhesions following IP injection, as compared to those 5–20 µm in diameter. Thus, it may be of future interest to obtain larger microspheres, centered in the 20–60 µm size range, for example by reducing the stirring speed of the 2nd emulsion step [13]. However, larger microspheres will have a reduced surface-to-volume ratio, likely resulting in a lower release rate. In parallel, it will also be necessary to incorporate a separation technique, such as sieving, in order to obtain monodispersed populations of microspheres. Alternately, well-defined microspheres may be obtained using membrane emulsification [33] or microfluidics [34]. 

**Figure 2:**
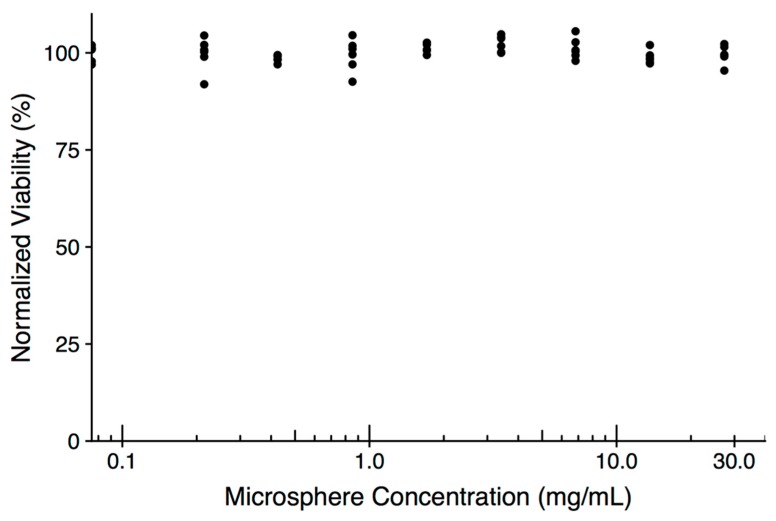
Normalized viability of L929 cells exposed to a range of microsphere concentrations for 48 h. Each dot represents a technical replicate; the experiment was repeated three times with similar results.

We screened for any potential cytotoxicity of empty microspheres (in the absence of drug), due to—for example—residual solvent. We performed a direct contact assay, based on ISO 10993-5 [35], using L929 murine fibroblasts incubated with a range of microsphere concentrations. After 48 hours of culture, no cytotoxicity was observed using resazurin viability assay, even for the highest concentration of microspheres (Figure 2).

Next, we assessed CPt-loaded microspheres. The loading efficiency of CPt was 72% ± 1% (n = 4 batches) and the encapsulated dose was calculated to be 25.4 ± 2.6 µg CPt per mg of polymer or 2.54% w/w (n = 4 batches). This is less than values reported in the literature, where encapsulated amount of platin ranged from 4% w/w [18] to as high as 40% w/w [14], but in those systems solid drug was dispersed in polymer—in the latter case the drug was clearly present as crystals. Here we are using a double emulsion process to yield CPt trapped inside a polymer membrane. A higher encapsulated dose could likely be achieved if the concentration of CPt in the aqueous phase was higher. We used a pharmaceutical CPt formulation at the highest dosage available, 10 mg/mL, but CPt is considered highly water soluble, with a predicted solubility above 70 mg/mL [36], thus several-fold higher encapsulation should be feasible. A typical platin chemotherapy regimen include 6 courses of doses in the range of 100–300 mg/m^2^ [5]. Based on an average body surface area of a female of 1.6 m^2^, the cumulative platin delivered can be estimated to be in the 1000–3000 mg range, requiring 40–120 g of microspheres, at the present encapsulation rate. While high, this is a feasible dose for IP injection as a dispersion in saline and could likely be further reduced 2–3 fold if a high concentration aqueous CPt formulation was used, increasing the encapsulated dose. 

**Figure 3:**
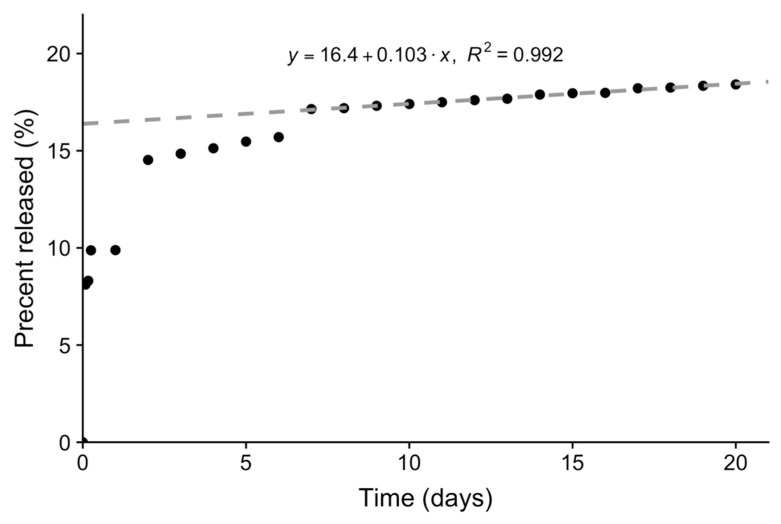
Carboplatin release from polymeric microspheres over 20 days in vitro with steady-state region fitted using a linear (zero-order) model (grey dashed line). Each dot represents the mean of 3 technical replicates.

To assess the in vitro release of CPt from microspheres, we used the spectrophotometric SnCl_2_ assay and monitored release over the course of 20 days (Figure 3).

The release profile showed an initial burst phase, with approx. Ten percent of the total loading of carboplatin being released from microspheres during first 24 h. Then, the system achieves steady state and a zero-order release was observed for the remainder of the 20-day experiment. Overall, these results are consistent with what would be expected for a diffusion-controlled reservoir system [27]. Here, the double emulsion process results in CPt being trapped inside a polymer membrane. The inner surface of the membrane, exposed to the excess CPt, can be expected to be saturated with CPt, while the 40 mL total volume of the receiving liquid and replacement after sampling ensure adequate sink conditions. Initially, due to the first step of the double emulsion process, it is likely the entire membrane is saturated with CPt, accounting for the burst release. Afterwards, zero-order kinetics (constant flux) will be maintained until the CPt concentration inside the microsphere drops below that sufficient to saturate the inner surface of the polymer membrane. In this case, the zero-order release is very slow (~0.1% of the encapsulated dose per day), due to the hydrolytic stability of the PET-DLA copolymer, as well as the presence of a boundary layer, which can be expected to add a significant diffusional resistance, given the relatively large size of the particles and poor mixing conditions inside the dialysis membrane. However, these conditions may more closely resemble the in vivo release scenario, where well-stirred conditions are unlikely. Overall, our results indicate much higher stability of these microspheres as compared to gelatin microspheres [25], from which 40% of the total carboplatin was released in first half an hour and the full dose was delivered over ~24 h, or to amphiphilic DTH-DLA-PEG2000 microspheres [28] from which 17% of loaded carboplatin was released during first 24 h, with the remainder being released over ~2 weeks. Importantly, current clinical practice involves chemotherapy regimens that last up to 32 weeks (>200 days), thus the slow zero-order release of CPt from our microspheres in vitro predicts promising in vivo performance.

**Figure 4:**
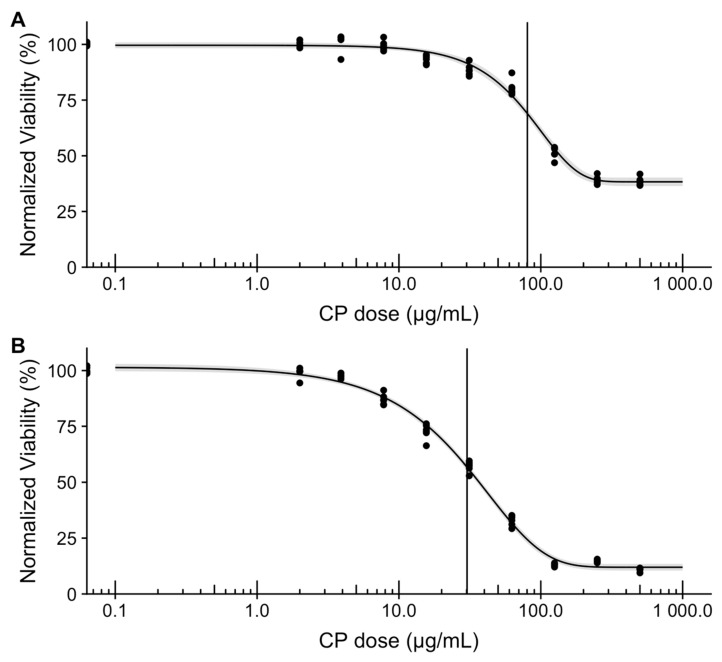
Human ovarian adenocarcinoma (SK-OV-3) cell susceptibility to CPt after exposure for 48 hours (**A**) or 72 h (**B**). Dots represent technical replicates (6), curve represents Weibull 4-parameter fit (grey band provides 95% confidence interval), and vertical bar marks the ED50 dose (80 µg/mL and 30 µg/mL for 48 h and 72 h, respectively).

Next, we assessed the in vitro efficacy of the microcapsules loaded with CPt against SK-OV-3 human ovarian adenocarcinoma cells. First, we confirmed the susceptibility of SK-OV-3 cells to CPt after 48-h and 72-h exposure to a range of CPt concentrations (Figure 4).

After 48 h, we observed ~40% viability of SK-OV-3 cells treated with the highest dose 500 µg/mL, indicating that the maximal effect was ~60% reduction in viability (Figure 4A). Based on Weibull 4-parameter fitting, we calculated the dose needed to achieve 50% of the maximal effect (ED50) to be 80 µg/mL. These results are in excellent agreement with those previously published [37]. After 72 h, as expected, greater potency is observed over the same CPt concentration range (Figure 4B), with the maximal effect being ~90% reduction in viability, yielding an ED50 of 30 µg/mL. Again, these results are in very good agreement with those previously published [38]. The 72-h exposure experiment was repeated and yielded similar results.

**Figure 5:**
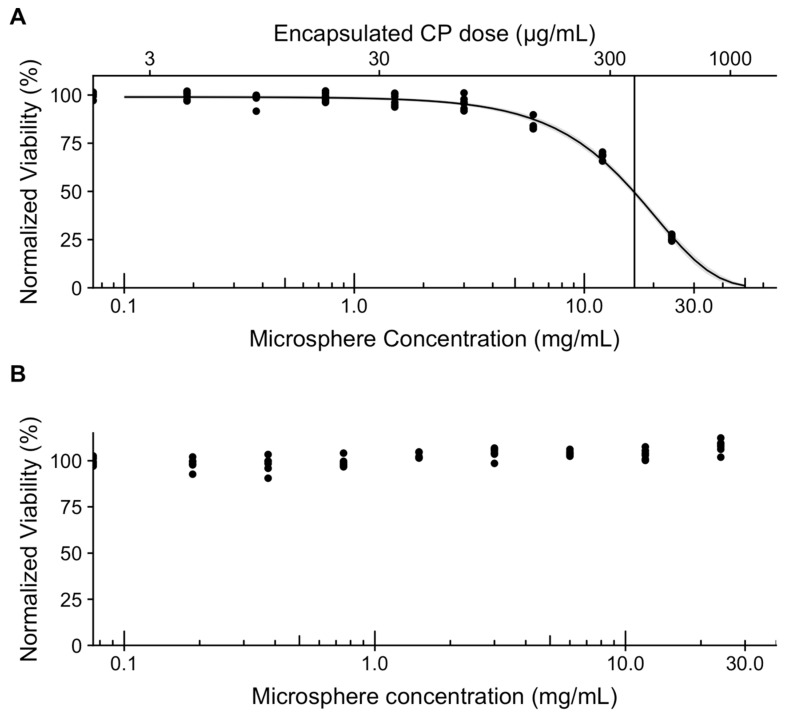
(**A**) Normalized viability of SK-OV-3 cells incubated for 72 h with carboplatin (CPt)-loaded microspheres. Bottom axis presents microsphere concentration, while the top axis presents the encapsulated CPt dose. Dots represent technical replicates (6 for each concentration), curve represents Weibull 3-parameter fit (grey band provides 95% confidence interval), and vertical bar marks the ED50 dose (17 mg/mL). (**B**) Normalized viability of SK-OV-3 cells incubated for 72 h with empty microspheres. Dots represent technical replicates (6 for each concentration).

Based on the data from the CPt susceptibility study, for evaluating the efficacy of CPt-loaded microspheres, we selected the 72-h exposure protocol, because the maximal effect was more pronounced and this time frame was a better fit to the initial release phase of our microspheres. After 72 h of incubation with CPt-loaded microspheres (total encapsulated CPt dose range ~6–600 µg/mL, as eight 2-fold serial dilutions) we observed a marked dose-dependent reduction in SK-OV-3 cell viability (Figure 5A), while no reduction in viability was observed for empty microspheres (Figure 5B), as for L929 cells.

At the highest microsphere concentration (24 mg/mL) we observed a ~75% reduction in SK-OV-3 cell viability and the ED50 value calculated from the Weibull 3-parameter fit was 17 mg/mL. Based on the CPt loading, this microsphere concentration would have an encapsulated CPt dose of 383 µg/mL. This ED50 value is >10 times greater than that obtained for 72-h exposure to free CPt, confirming that the loaded CPt is not released as a bolus, but rather over an extended period of time, as designed. The difference in ED50 values is consistent with the in vitro CPt release profile (Figure 3): Based on the area-under-the-curve of first 72 h of the release profile, approx. ~12% of the encapsulated dose would be released—and thus bioavailable—in an ideal scenario of sink conditions in water. Thus, the ED50 value obtained experimentally under in vitro cell culture conditions (serum-containing media) is very reasonable. The experiment was repeated 3 times with similar results. We conclude that CPt-loaded microspheres are capable of releasing CPt that is bioavailable and results in reduced viability of SK-OV-3 human ovarian adenocarcinoma cells in vitro. Importantly, the dose response observed for CPt-loaded microspheres in the cell culture cytotoxicity study indicates that the sustained release profile was not markedly altered by the presence of serum.

Based on the encouraging in vitro cell culture results and in vitro release profile, we proceeded to test our CPt-loaded microspheres in vivo, focusing on the question of CPt release and bioavailability. Equivalent CPt doses (1 mg), as neat CPt or CPt-loaded microspheres (~39 mg of microspheres encapsulating 1 mg of CPt), were injected IP into mice (14 mice per group). All mice survived intraperitoneal injection of drug or microspheres and all 28 mice completed the study. The behavior and weight of all animals were monitored for a total of 14 days. We did not observe any differences in feeding behavior (such as eating delay) in either group, during the entire experiment. Likewise, the amount of food consumed by mice within 5 min (serving as a control of appetite change) did not change throughout the experimental period. At the end of the experimental period, the average weight was 20.8 g, which did not differ from the initial weight (20.5 g). Overall, the behavior of all of the mice during eating and resting did not raise any suspicion of aggression or stress. In summary, there were no changes in mouse behavior during the whole experiment and no differences between groups.

**Figure 6:**
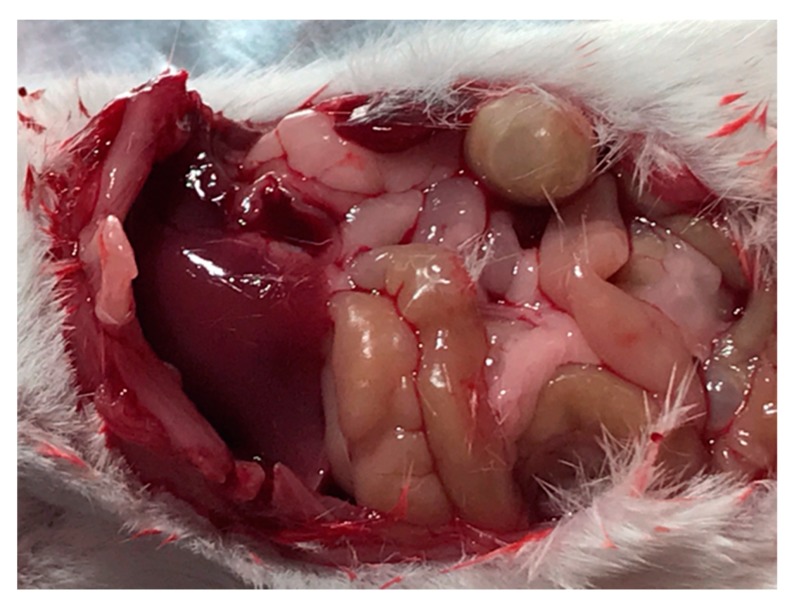
Photograph of abdominal cavity following gross dissection, 7 days after intraperitoneal (IP) injection of microspheres encapsulating CPt.

At each time point, a pair of animals from each group sacrificed (6 h, 12 h, 1 day, 2 days, 3 days, 7 days, 14 days). Following sacrifice, blood samples were collected and the abdominal cavities were dissected to collect tissue samples. In Figure 6 we present a representative photo of the abdominal cavity following sacrifice and gross dissection of a mouse in the experimental group, 7 days after receiving IP injection of CPt-loaded microspheres. Importantly, during abdominal dissection, we did not note the formation of adhesions, pathological bonds between intra-abdominal organs and/or the peritoneal wall [39]. Adhesions are caused by peritoneal injury or presence of foreign bodies, for example powder from surgical gloves or lint from surgical drapes, and are a major source of complications following gastrointestinal surgery [39]. As a result, adhesion formation is a concern for any DDS injected IP. Previously, Kohane et al. reported that IP administration of PLGA microparticles >5 µm in diameter in mice resulted in the formation of adhesions [32]; however, they also observed that larger microparticles, 20–60 µm in diameter, resulted in reduced adhesions, as compared to those 5–20 µm in diameter. Approx. 40% of our microspheres fall within the 20–60 µm size range, while our injected dose (~39 mg) was within the dose range (10–100 mg) Kohane et al. tested. As a result, it may be important to consider shifting the median of our microsphere distribution into this larger size range. Meanwhile, the role of dosage will also require further investigation, because for the 60-µm microparticles, Kohane et al. observed fewer adhesions with increasing injected particle mass, with none observed for the 100 mg dose (estimated to be equivalent to ~35 g in a human) [32]. However, this is encouraging from the point of view of scale up to clinically-relevant dosages for human patients.

**Figure 7:**
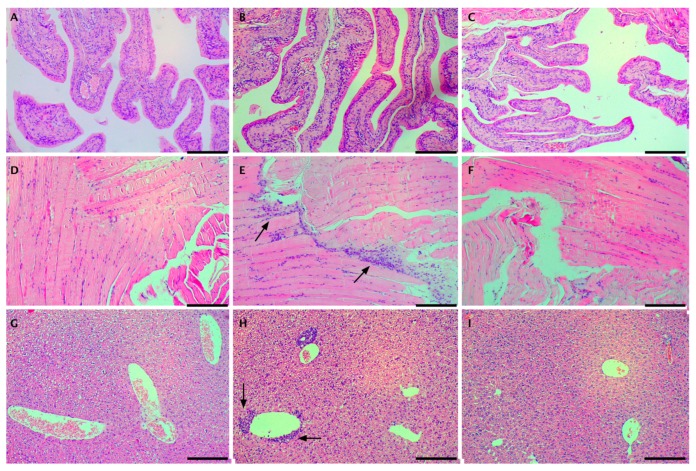
Representative micrographs of H&E stained tissue specimens. Panels **A** (ovary), **D** (parietal peritoneum), **G** (liver) are from a control animal (neat CPt) after 6 h. Panels **B** (ovary), **E** (parietal peritoneum), **H** (liver) are from an experimental animal (CPt HS) after 6 h. Panels **C** (ovary), **F** (parietal peritoneum), **I** (liver) are from an experimental animal (CPt HS) after 14 days. Scale bar represents 200 µm; arrows indicate presence of moderate inflammatory infiltrate.

From each group, at each time point, the following tissues from one animal were stained with hemotoxylin and eosin (H&E) for histological examination: Visceral peritoneum, parietal peritoneum, ovary, and liver. Representative micrographs are presented in Figure 7. For the case of the control group, receiving neat CPt, neither abnormalities nor inflammatory infiltrates were observed in any of the tissues, at any time point. For the case of the experimental group receiving microspheres with encapsulated CPt, no histopathological changes or abnormalities were observed in any specimens of the visceral peritoneum and ovary. However, we did observe signs of inflammation in 3 specimens of parietal peritoneum: A focal purulent infiltrate at the 6 h time point, a small neutrophil cluster loosely present on the peritoneal surface at the 12 h time point, and a moderate intensity lymphoid infiltrate at the 7 day time point. In liver specimens, we observed 2 cases of inflammatory infiltration around the gallbladder spaces (6 h and 12 h), as well as scattered clusters of lymphoid cells in three specimens at the later time points (2 days, 3 days, 7 days, 14 days).

Overall, the histological results are consistent with mild, clinically-insignificant local inflammatory response to the introduction of microspheres into the peritoneal cavity. Importantly, in contrast to the observations of Kohane et al. [32], following IP injection of PLGA microspheres in mice, we did not observe any foreign body giant cells. Likewise, we did not observe any foamy macrophages along the injection track, only isolated lymphocytes and neutrophils.

**Figure 8:**
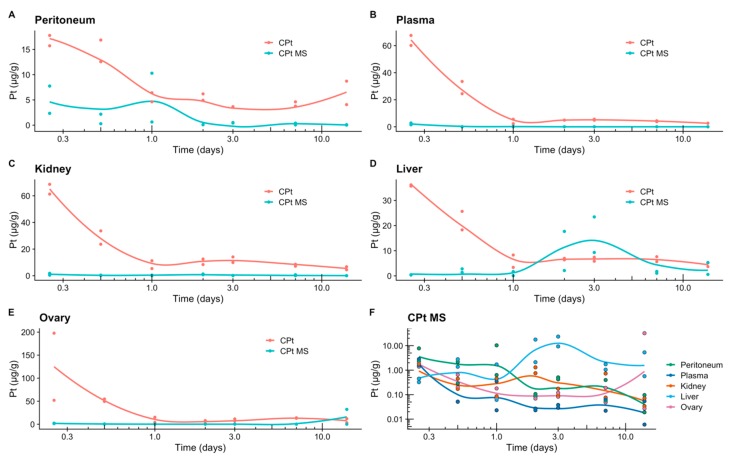
Tissue platinum distribution over time, following one time injection of 1 mg of free CPt (CPt, red) or 1 mg of CPt encapsulated in microspheres (CPt MS, teal). Panel F presents summary of data for the experimental animals that received CPt MS. Each point represents one animal sacrificed at the given time point. To aid the eye, curves present locally estimated scatterplot smoothing (LOESS).

Finally, in order to examine the in vivo release of CPt from microspheres, we assayed for elemental platinum, as a proxy for CPt, using flame atomization (Figure 8), in collected blood samples, as well as tissue samples from the liver, peritoneum, ovaries, and kidney. As expected, IP injection of neat CPt resulted in high platinum concentration detected in plasma, as well as in the ovary, kidney, and liver, while platinum levels in the peritoneum were lowest. This confirms the rapid absorption of the hydrophilic, small molecule CPt into the systemic circulation, an effect has also been observed in human trials [7]. In contrast, in the plasma and tissues of animals receiving CPt encapsulated in microspheres platinum levels were markedly lower and we did not observe a major burst effect. This confirms that the entire encapsulated dose is not immediately bioavailable, but rather released over time. As a result, platinum levels in the ovary were also markedly lower than those in animals receiving neat CPt. However, after 14 days, when the hypothetical patient would be preparing for another course of chemotherapy, IP injection of neat CPt resulted in ~10 fold higher platinum levels in the kidney and plasma compared to the same dose of CPt encapsulated in microspheres. Thus, we conclude that using the encapsulation system, a much higher encapsulated dose—at least 5 times greater—could potentially be safely delivered, resulting in higher sustained levels of platinum in the ovary. Thus, it is plausible that 6 courses of current standard-of-care platin IP infusion therapy could be converted to a one-time IP injection of microspheres.

In comparison to work with PLA microspheres encapsulating cisplatin by Tamura et al. [23], we observed approx. 2 times lower levels of platinum in the kidney at all time points, despite injecting approx. twice the encapsulated platin dose. However, for our system, the platinum levels in the liver rise to higher values 2–3 times higher than those reported by Tamura et al. In fact, 3 days after injection, the microspheres resulted in higher platinum levels in the liver than IP injection of neat CPt. Further studies will be needed to clarify any potential risks associated with this effect.

## 3. Materials and Methods

### 3.1. Chemicals and Reagents

Segmented copolymer was prepared using dimethyl terephthalate for synthesis (DMT, ≥99.0%) (Merck, Darmstadt, Germany), monoethylene glycol (EG, ≥99.5%) (Brenntag, Poland) and dimer linoleic acid, abbreviated as dilinoleic acid (DLA), trade name Pripol 1009 (hydrogenated, distilled dimer acid 98%, molecular mass ~570 g·mol^−1^ (C36)), kindly provided by Croda (Gouda, The Netherlands). All solvents (chloroform, propanol, methanol, and anhydrous methylene chloride) of analytical (HPLC) grade were purchased from POCH (Gliwice, Poland) and used without further purification. Carboplatin (CPt) was in the form of aqueous concentrate for infusion (10 mg/mL, Carbomedac, medac GmbH). Poly(vinyl alcohol) (PVA) of molecular mass of 13–23 kDa (87–98% hydrolysed) was purchased from Sigma Aldrich (Poznań, Poland). All cell culture reagents were purchased from Sigma Aldrich (Poznań, Poland). All chemicals and reagents were used as received and stored following the manufacturers’ instructions.

### 3.2. Polymer for Drug Encapsulation

Poly(ethylene terephthalate-ethylene dilinoleate) (PET-DLA) was synthesized via a two-stage process of transesterification and polycondensation from the melt as described previously [40,41]. Briefly, the transesterification was carried out in the presence of monoethylene glycol, and dimethyl terephthalate at 180 °C, until 95% of methanol was collected. Before the polycondensation step, DLA was added to the reaction mixture, the pressure was decreased to 0.2–0.4 hPa, and the temperature was increased to 260 °C. The progress of the polycondensation reaction was followed by monitoring the power consumption of stirrer. Once a constant value of power consumption by the stirrer was observed, the material was extruded from reactor into cold water and material thread was pelletized.

**Figure 9:**
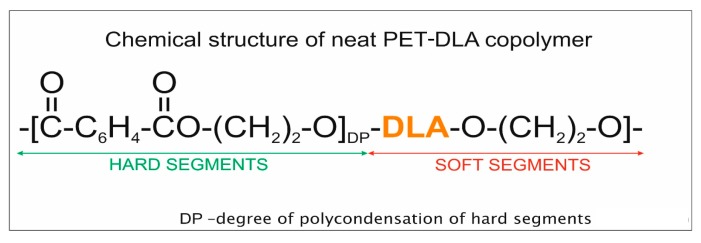
Chemical structure of poly(ethylene terephthalate-ethylene dilinoleate) (PET-DLA) copolymer used for microspheres preparation.

The chemical structure of PET-DLA copolymer containing 40 wt.% of hard segments of ethylene terephthalate and 60 wt.% of ethylene dilinoleate is shown in Figure 9.

Detailed characterization of the PET-DLA copolymer can be found in our previous work [42], e.g., differential scanning calorimetry (DSC): T_m_ = 166.2 °C, T_g_ = −19.1 °C, degree of crystallinity of hard segments, X_c_ = 8%.

### 3.3. Preparation of Microspheres

**Figure 10:**
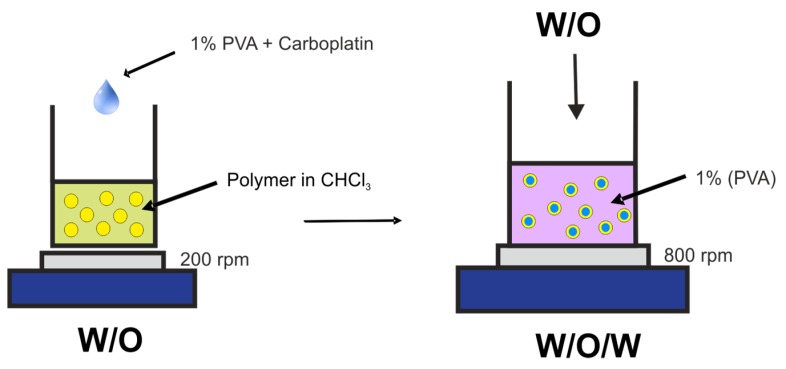
Double emulsion process for obtaining PET-DLA microspheres encapsulating carboplatin.

Microspheres were obtained *via* simple double emulsification process (Figure 10), based on our previous work [28], under sterile conditions. Briefly, an aqueous solution of the CPt (200 µL, 10 mg/mL) was mixed with 100 µL solution of 1% PVA in a distilled water, followed by emulsification in 1 mL of polymer solution (40 mg in 1 mL of chloroform) by drop-wise addition while magnetic stirring at 400 rpm for 2 min. This formed the primary (water/oil) emulsion. Next, the primary emulsion added drop-wise to 10 mL of 1% PVA in a distilled water, while stirring at 800 rpm over the course of 4 min, followed by 5 min of additional stirring, thus forming the secondary (water/oil/water) emulsion. Finally, the stirring was reduced to 400 rpm and the chloroform was removed by evaporation over 24 h in a fume hood at room temperature, while protected from exposure to light. After evaporation of the solvent, the resulting microspheres were centrifuged (2500 RCF for 10 min) and washed with deionized water 3 times. The resulting product was lyophilized using a laboratory freeze dryer (Christ Alpha 1–2 LD plus). Empty microspheres were prepared in the same fashion, but without addition of CPt. Lyophilized microspheres were stored refrigerated and redispersed on-demand in the desired liquid (saline, media, etc.) using a lab vortex.

### 3.4. Morphology of Microspheres

To assess the morphology and determine size of obtained microspheres, a drop of freshly prepared microspheres dispersed in distilled water was placed on a microscope slide, covered with a cover slip, and imaged using optical microscope (Delta Optical IB-100) at 10× magnification. Image analysis was performed using Fiji software [43].

### 3.5. Loading and Release of Carboplatin In Vitro

Carboplatin loading efficiency was determined by assaying the supernatant after initial centrifugation using SnCl_2_ assay [44] and comparing this un-encapsulated amount to initial amount of carboplatin (feed). Briefly, 1 mL of test solution was mixed with 1 mL of 1 mM SnCl_2_ in 1 N HCl and incubated for 50 min at room temperature protected from light. Carboplatin concentration was then determined by measuring absorbance at 398 nm (Jasco V-630 PC spectrophotometer) and comparing to a standard curve prepared from samples of known concentration. 

To assess in vitro release of carboplatin from microspheres, ~20 mg of microspheres were dispersed in 10 mL of distilled water and transferred to a dialysis membrane with molecular MWCO cut-off (separation limit) of 6000–8000 kDa (Spectra/por 1). The dialysis membrane was then placed in a beaker filled with 30 mL of distilled water and incubated with rocking motion at 37 °C for 20 days. The release of carboplatin was monitored by assaying 1 mL samples using the SnCl_2_ assay, as described above. At each time point, 3 samples were collected and the appropriate volume of fresh distilled water was replaced into the breaker. The three measurements were averaged and plotted as a function of time. After the burst phase, the remaining data was fitted to a zero-order model using the *lm* function in R software (RStudio).

### 3.6. In Vitro Assessment of Microsphere Cytotoxicity and Drug Delivery

Cytotoxicity study of empty microspheres was performed by the direct contact assay, based on ISO 10993-5 [35], using L929 murine fibroblasts (Sigma Aldrich, Poznań, Poland). Briefly, in a 96-well plate, 5000 L929 cells were seeded in 100 µL of Dulbecco’s Modified Eagle Medium (DMEM) containing 10% fetal bovine serum (FBS), 2 mM L glutamine, 100 U/mL penicillin, and 100 µg/mL streptomycin (referred to further as “complete DMEM”). After 24 h of culture, media was replaced for media containing 2-fold serial dilutions of microspheres resuspended in complete DMEM (range: 0.2 to ~30 mg/mL). After further 48 h of incubation, cell viability was assessed using resazurin assay [45]. Briefly, the media was replaced with complete media containing 0.025 mg/mL resazurin and the plate was incubated for 4 h at 37 °C, followed by fluorescence measurement (Em: 540, Ex: 590) using BioTek Synergy HTX multifunctional plate reader.

For in vitro assessment of drug delivery from microspheres loaded with CPt, we used SK-OV-3 human ovarian adenocarcinoma cells (Sigma Aldrich, Poznań, Poland). Cells were maintained in McCoy’s 5a media containing 15% fetal bovine serum (FBS), 2 mM L glutamine, 100 U/mL penicillin, and 100 µg/mL streptomycin (referred to further as “complete McCoy’s media”). Based on NCI60 protocol [46], we first confirmed SK-OV-3 susceptibility to CPt. Briefly, in 96-well plates 10,000 SK-OV-3 cells per well were seeded in 100 µL of complete McCoy’s media and incubated for 24 h, after which the media was replaced with complete McCoy’s media containing serial 2-fold dilutions of CPt, spanning a range of concentrations from 500 µg/mL to 2 µg/mL. After 48 h of incubation, cell viability was assessed using microscopy and the resazurin assay [45], as described previously. Additionally, we performed a 72-h variant of the experiment, by seeding 5000 SK-OV-3 cells per well and incubating for 72 h with the same range of CPt.

To test microspheres loaded with CPt, we used a 72-h incubation variant of the direct contact assay described previously. Briefly, in 96-well plates, 5000 SK-OV-3 cells per well were seeded in 100 µL of complete McCoy’s media and incubated for 24 h, after which the media was replaced with complete McCoy’s media containing eight 2-fold serial dilutions of microspheres (range: ~0.2 to ~30 mg/mL), spanning a range of encapsulated CPt concentrations from ~600 µg/mL to ~6 µg/mL. Following additional 72 h of incubation, cell viability was assessed using microscopy and the resazurin assay [45], as described previously. As a control, empty microspheres were used. Dose response data was analyzed in R software (RStudio) using *drc* package [47].

### 3.7. In Vivo Assessment of Drug Delivery

Female Balb C nude mice, 28 weeks old, were purchased from Anima Lab (Poznan, Poland). The use of mice and their treatment was approved by Ethical Committee for Animal Experiments decision no. 1/215, and all the experiments were carried out in strict compliance with regulations. The animals had a genetic certificate and a health certificate issued by a veterinarian. The animals were housed 6 to a cage, at the animal facility of the Pomeranian Medical University, Szczecin. The air humidity in the animal room was approx. 55%, the temperature was maintained at 22 ± 2 °C, and a 12/12 light cycle was controlled by an automated system. Before the study, all animals were weighed; their average body weight was 20.5 g. The animals were fed Labofeed H feed (protein: 22.9%, fat: 4.1%, fiber: 4.6%) for 2 weeks, with a caloric content of 35 kcal per day, while water access was ad libitum. This time served to adapt the animals to the new conditions and permit observation. All animals survived the adaptation period. The experiments were conducted with the use of pharmaceutical form of CPt (Pfizer) and suspension of microcapsules made of PET-DLA polyester microspheres containing CPt, which was released in a controlled manner. The CPt dosage was developed on the basis of available literature data [48,49]. Two groups were studied: 14 mice were given 1 mL of liquid containing 1 mg of CPt pharmaceutical formulation (10 mg/mL) via IP injection, while another 14 mice were given 1 mL of saline with a dispersion of microspheres containing the same CPt dose (~39 mg of microspheres encapsulating 1 mg CPt), also via IP injection.

All mice survived intraperitoneal administration of drugs and all 28 mice completed the study. The health and weight of all animals were monitored daily for a total of 14 days, with a pair of animals from each group sacrificed at each time point (6 h, 12 h, 1 day, 2 days, 3 days, 7 days, 14 days). Forane isoflurane (from AbbVie) was used for anesthesia and euthanasia due to its speed of action, lack of stress, and extrahepatic metabolism. Isoflurane was supplied in the form of gas to a special chamber. The chamber was prepared so that it could ensure proper distribution of gas and fast exposure of the mouse to its concentration. The animals were initially exposed to an anesthetic isoflurane dose for blood collection, followed by a lethal dose. The death of each animal was determined on the basis of cardiac arrest. In addition to blood samples, tissue samples were collected from the liver, peritoneum, ovaries, and kidney. All samples were frozen.

### 3.8. Release of Carboplatin In Vivo

The concentration of Pt released into plasma and tissues (liver, peritoneum, ovaries, kidney) was determined by a Shimadzu AAS 7000 spectrometer (Shimadzu, Japan) with flame atomization (F-AAS). All samples were lyophilized prior to processing (Christ Alpha 1–2).

## 4. Conclusions

In this work we demonstrated a double emulsion process for obtaining CPt-loaded microcapsules composed of poly(ethylene terephthalate-ethylene dilinoleate) (PET-DLA) copolymer. The obtained microcapsules were in the target size range of 10–25 µm, in order to reduce intraperitoneal clearance by phagocytosis and lymphoid transit. Importantly, empty microspheres did not exhibit toxicity in vitro. The double emulsion process yielded 2.5% w/w CPt loading and, in contrast to previous work, the obtained microcapsules exhibited long-term sustained (>20 day) zero-order release. The released CPt was confirmed to be bioavailable, as CPt-loaded microcapsules demonstrated efficacy against human ovarian adenocarcinoma (SK-OV-3) cells in vitro. Following intraperitoneal injection in mice, tissue platinum levels revealed low burst and reduced systemic uptake (plasma, kidney), as compared to neat carboplatin injection. Importantly, in contrast to previous work, we did not observe adhesions, only a mild, clinically-insignificant, local inflammatory response. Collectively, our results demonstrate the potential of the developed microencapsulation system for long-term intraperitoneal sustained release of carboplatin, as a part of ovarian anticancer therapy. The results presented here motivate further preclinical studies in animals to assess efficacy, for example in a xenograft model, focusing on ovarian cancer metastasis to the peritoneal surfaces.
